# Frequency and Significance of *Ras, Tert* Promoter, and *Braf* Mutations in Cytologically Indeterminate Thyroid Nodules: A Monocentric Case Series at a Tertiary-Level Endocrinology Unit

**DOI:** 10.3389/fendo.2017.00273

**Published:** 2017-10-16

**Authors:** Simona Censi, Elisabetta Cavedon, Loris Bertazza, Francesca Galuppini, Sara Watutantrige-Fernando, Paola De Lazzari, Davide Nacamulli, Gianmaria Pennelli, Ambrogio Fassina, Maurizio Iacobone, Eric Casal Ide, Federica Vianello, Susi Barollo, Caterina Mian

**Affiliations:** ^1^Endocrinology Unit, Department of Medicine (DIMED), University of Padova, Padova, Italy; ^2^Surgical Pathology and Cytopathology Unit, Pathology Unit, Department of Medicine (DIMED), University of Padova, Padova, Italy; ^3^Endocrine Surgery Unit, Department of Surgical, Oncological and Gastroenterological Sciences (DiSCOG), University of Padova, Padova, Italy; ^4^Department of Radiotherapy, Istituto Oncologico Veneto-IRCCS, Padova, Italy

**Keywords:** thyroid nodules, indeterminate thyroid cytology, *TERT* promoter mutations, *H-,K-,N-RAS* mutations, *BRAF* mutations

## Abstract

**Purpose:**

The management of thyroid nodules of indeterminate cytology is controversial. Our study aimed to establish the frequency and significance of *H-,K-,N-RAS, TERT* promoter, and *BRAF* gene mutations in thyroid nodes of indeterminate cytology and to assess their potential usefulness in clinical practice.

**Methods:**

*H*-,*K*-,*N-RAS, TERT* promoter and *BRAF* gene mutations were examined in a series of 199 consecutive nodes of indeterminate cytology referred for surgical excision.

**Results:**

69/199 (35%) were malignant on histopathological review. *RAS* mutations were detected in 36/199 (18%), and 19/36 cases (53%) were malignant on histological diagnosis. *TERT* promoter mutations were detected in 7/199 (4%) nodules, which were all malignant lesions. *BRAF* mutations were detected in 15/199 (8%), and a *BRAF* K601E mutation was identified in 2 follicular adenomas and 1 noninvasive follicular thyroid neoplasm with papillary-like nuclear features. Altogether, this panel was able to identify 48% of the malignant lesions, achieving a specificity, positive predictive value, and negative predictive value for malignancy of 85, 62, and 75%, respectively.

**Conclusion:**

The residual malignancy risk in mutation-negative nodes is 25%. These nodes still need to be resected, but mutation analysis could help to orient the appropriate surgical strategy.

## Introduction

Fine-needle aspiration (FNA) cytology is the gold standard for the nonsurgical assessment of thyroid nodules, but it is unable to exclude cancer in one in four cases, which are labeled as “indeterminate” (1). According to the Italian Society of Anatomic Pathology and Cytology (SIAPEC) Consensus Statement, this “indeterminate” category is very heterogeneous, including cytological patterns that range from follicular types and Hürtle cell lesions to scant atypical features. The cancer risk varies, reportedly averaging around 26% in the largest series (2, 3). A proposed updated classification divides atypical cellular lesions into two categories, as explained in the SIAPEC 2014 Consensus Statement (4). Indeterminate lesions are separated into TIR 3A and TIR 3B, with an estimated malignancy risk below 10% for the former, and around 15–30% for the latter. The studies published to date are not enough to confirm these malignancy risk predictions, so there is still uncertainty regarding the clinical approach to such nodules. To orient patient management, reference might be made to the malignancy risk of the extensively studied Bethesda classification. Any comparisons drawn between the indeterminate categories of the Bethesda and SIAPEC classifications should be considered with caution, however, for several reasons. For instance, atypia and follicular lesions of undetermined significance (AUS/FLUS) also includes cytological/nuclear atypia in the Bethesda system, which would convert a TIR 3A into a TIR 3B in the SIAPEC system (5). Such an unclear picture sometimes makes it difficult to manage patients with thyroid nodules of indeterminate cytology and to choose between surveillance and diagnostic surgery. In fact, most of such patients undergo diagnostic surgery, but only a minority of their surgically resected nodules prove to be malignant (6). Thyroid lobectomy can be performed for solitary nodules, but then patients need a second surgical procedure to complete the thyroidectomy if histology identifies them as malignant. A more accurate preoperative diagnosis of cancer in thyroid nodules would enable such unnecessary or two-step surgery to be avoided.

In this clinical setting, molecular profiling has been suggested as a way to diagnose nodules of indeterminate cytology more accurately (7), and rule cancer in or out in the light of the test’s positive predictive value (PPV) or negative predictive value (NPV), respectively.

Among the various molecular testing approaches, searching for *BRAF* and *H-, K-*, and *N-RAS* point mutations is currently the most widely used for thyroid nodules of indeterminate cytology. *BRAF* point mutations are the most common gene mutations in thyroid cancer, occurring in 45% of papillary thyroid carcinomas (PTC), 20–40% of poorly-differentiated thyroid carcinomas (PDTC), and 30–40% of anaplastic thyroid carcinomas (ATC) (8, 9), while they are rarely seen in follicular thyroid carcinomas (FTC). *BRAF* V600E mutation testing has a high specificity for malignancy, but its sensitivity is too low to rule out cancer reliably (10). Mutational analysis therefore needs to consider other mutations too, such as *H-RAS, K-RAS*, and *N-RAS* point mutations. *RAS* mutations occur in 10–20% of PTC (11), 36–48% of FTC, and 20–40% of PDTC and ATC (10), but they are not specific for thyroid malignancy and they also occur in follicular adenoma (FA).

Telomerase activation is considered a hallmark of cancer because it is frequently activated in many human cancers, but not expressed in most normal tissues. TERT promoter mutations have recently been described in thyroid cancers deriving from follicular cells (12), occurring in 11% of PTC, 17% of FTC, 43% of PDTC, and 40% of ATC (13). Thyroid tumors carrying *TERT* promoter mutations generally have more aggressive characteristics and higher tumor recurrence and mortality rates (13). *TERT* promoter mutations therefore have potential as novel diagnostic and prognostic markers in the setting of thyroid cancer.

Our study aimed (1) to establish the frequency and significance of *RAS, TERT* promoter, and *BRAF* gene mutations in a large series of patients undergoing thyroid surgery for a nodule of indeterminate cytology and (2) to examine the potential utility of these mutations in clinical practice.

## Materials and Methods

### FNA Samples and FNA Procedures

The study involved 199 FNA samples of indeterminate cytology obtained from thyroid nodules in 199 consecutive patients referred for surgical excision from December 2012 to May 2016. At our institution, *BRAF* and *H-, K-*, and *N-RAS* mutation analysis in FNA samples is standard procedure for single thyroid nodules and/or those with suspect features on ultrasonography. Molecular analysis for somatic mutations of *TERT* promoter was performed retrospectively. All studies were performed in accordance with the guidelines proposed in the Declaration of Helsinki. The present study was notified to our Local Ethical Committee (Azienda Ospedaliera di Padova, code number: 42109) and all patients (including the parent/guardian on behalf of the minors) gave their written informed consent to the use of their thyroid cytology findings for research purposes. In this sample of patients, 165/199 (83%) underwent total thyroidectomy, 25/199 (13%) had a lobectomy, and 9/199 (4%) a two-step thyroidectomy.

Of the 199 cases collected, the first 88 were classified according to the SIAPEC 2007 classification, and the remainder (111 cases) according to the revised version published during the course of the study (SIAPEC 2014) (4).

### *BRAF* and *RAS* Mutation Analysis

DNA was isolated from FNA samples using the QIAamp DNA Micro kit (Qiagen, Italy) according to the manufacturer’s protocol. The *BRAF* (NM_004333.4) status of exon 15 was assessed by direct sequencing. The primers and PCR reaction protocol have been described elsewhere (14). Exons 2 and 3 of *H-RAS* (NM_005343.2), *K-RAS* (NM_033360.2), and *N-RAS* (NM_002524.3) were analyzed by direct sequencing, as described elsewhere (15, 16).

### *TERT* Promoter Analysis

The *TERT* proximal promoter (NM_198253.2) was amplified from FNA samples using the amplification conditions described elsewhere (17).

### Statistics

Sensitivity, specificity, PPV, and NPV were calculated for each mutation, and for combined methods, considering the histological diagnosis as the gold standard. Group comparisons of categorical variables were performed using the chi-square test. A *p*-value of <0.05 was considered significant in all tests. All statistical analyses were performed using MedCalc for Windows, version 17.6.

## Results

### Patient Statistics

Of the 199 patients included in our study, 155 (78%) were female and 44 (22%) were male. The mean age of the patients was 50 years (median 49 years). Among the 199 indeterminate fine-needle aspirates, 69 (35%) were classified as malignant on histopathological review (final histologies are given in Table 1). In the group of 111 patients classified according to the SIAPEC 2014 system, 63 were TIR 3A and 48 were TIR 3B. Fourteen out of 63 cases (22%) in the TIR 3A group, and 28/48 (58%) in the TIR 3B group proved malignant at histology. A significant correlation emerged between malignancy and a TIR 3B diagnosis (*p*: 0.004 by chi-square analysis).

**Table 1:** Final histological classification of 199 cytologically indeterminate nodules.

	Histological result	Numerosity
Benign: *n* = 130/199 (65%)	FA	87
	HN	29
	WDT-UMP	6
	NIFTP	8

Malignant: *n* = 69/199 (35%)	CV-PTC	24
	FV-PTC	14
	FTC-MI	16
	FTC-WI	13
	PTC/FTC	1
	MTC	1

*FA, follicular adenoma; FTC-MI, minimally invasive follicular thyroid carcinoma; FTC-WI, widely invasive follicular thyroid carcinoma; HN, hyperplastic nodule; CV-PTC, classical variant of papillary thyroid carcinoma; FV-PTC, follicular variant of papillary thyroid carcinoma; MTC, medullary thyroid cancer; NIFTP, noninvasive follicular thyroid neoplasm with papillary-like nuclear features; PTC/FTC, mixed papillary thyroid carcinoma and follicular thyroid carcinoma; WDT-UMP, well-differentiated tumor of uncertain malignant potential*.

### *RAS* Mutation Testing

*RAS* mutations were the most common, detected in 36/199 (18%) patients, and occurring in 28% (19/69) of the malignant nodules. Six nodules revealed an *H-RAS* mutation at codon 61, and one at codon 13. Three nodules carried a *K-RAS* mutation at codon 61, and two at codon 12. There were also 24 nodules with an *N-RAS* mutation at codon 61 (Table 2). Among the 36 patients exhibiting a somatic *RAS* mutation, 19/36 (53%) were malignant at histology (Table 3). Table 3 shows the sensitivity, specificity, PPV, and NPV for malignancy of these RAS mutations.

**Table 2:** Histological results in fine-needle aspirates from mutated nodules.

	*BRAF* deletion^a^	*BRAF* V600E	*BRAF* K601E	*H-RAS*, codon 13	*H-RAS*, codon 61	*K-RAS*, codon 12	*K-RAS*, codon 61	*N-RAS*, codon 61	*TERTp*C250T	*TERTp*C228T	*N-RAS* codon 61 + *TERTp* C228T	*K-RAS* codon 12 + *TERT*p C228T	*BRAF* K601E + *TERT*p C250T
FA	0	0	2	0	1	1	1	8	0	0	0	0	0
WDT-UMP	0	0	0	0	2	0	0	2	0	0	0	0	0
NIFTP	0	0	1	0	0	0	0	2	0	0	0	0	0
CV-PTC	1	8	0	1	1	0	2	2	0	0	0	0	0
FV-PTC	0	1	0	0	1	0	0	3	0	0	2	1	0
FTC-MI	0	0	0	0	0	0	0	1	0	0	0	0	0
FTC-WI	0	0	0	0	1	0	0	2	1	1	1	0	1
PTC/FTC	0	0	0	0	0	0	0	1	0	0	0	0	0

*FA, follicular adenoma; FTC-MI, minimally invasive follicular thyroid carcinoma; FTC-WI, widely invasive follicular thyroid carcinoma; CV-PTC, classical variant of papillary thyroid carcinoma; FV-PTC, follicular variant of papillary thyroid carcinoma; NIFTP, noninvasive follicular thyroid neoplasm with papillary-like nuclear features; PTC/FTC, mixed papillary thyroid carcinoma and follicular thyroid carcinoma; TERTp, TERT promoter; WDT-UMP, well-differentiated tumor of uncertain malignant potential*.

*^a^Triplet deletion c.1799_1801delTGA (p.V600_K601>E) without frame-shift*.

**Table 3:** Frequency, sensitivity, specificity, positive predictive value (PPV), negative predictive value (NPV), and histological correlations of *H-,K-,N-RAS, TERT* promoter, and *BRAF* mutations.

	Malignant histology (*n* = 69/199, 35%)	Benign histology (*n* = 130/199, 65%)	
*RAS* (*n* = 36/199, 18%)	*n* = 19/36, 53% 6 CV-PTC 7 FV-PTC 1 PTC/FTC 4 FTC-WI 1 FTC-MI	*n* = 17/36, 47% 11 FA 4 WDT-UMP 2 NIFTP	Sensitivity: 28% Specificity: 87% PPV: 53% NPV: 69%

*TERT* promoter (*n* = 7/199, 4%)	*n* = 7/7, 100% 3 FV-PTC 4 FTC-WI	*n* = 0/7, 0%	Sensitivity: 10% Specificity: 100% PPV: 100% NPV: 68%

*BRAF* (*n* = 15/199, 8%)	*n* = 12/15, 80% 9 CV-PTC 2 FV-PTC 1 FTC-WI	*n* = 3/15, 20% 2 FA 1 NIFTP	Sensitivity: 17% Specificity: 98% PPV: 80% NPV: 69%

*BRAF K601E* (*n* = 4/199, 2%)	*n* = 1/4, 25% 1 FTC-WI	*n* = 3/4, 75% 2 FA 1 NIFTP	Sensitivity: 1% Specificity: 98% PPV: 25% NPV: 65%

*BRAF* mutations other than *K601E* (*n* = 11/199, 6%)	*n* = 11/11, 100% 9 CV-PTC 2 FV-PTC	*n* = 0/11, 0%	Sensitivity: 16% Specificity: 100% PPV: 100% NPV: 69%

Any mutation (*n* = 53/199, 27%)	*n* = 33/53, 62%	*n* = 20/53, 38%	Sensitivity: 48% Specificity: 85% PPV: 62% NPV: 75%

*FA, follicular adenoma; FTC-MI, minimally invasive follicular thyroid carcinoma; FTC-WI, widely invasive follicular carcinoma; PTC, papillary thyroid carcinoma; CV-PTC, classical variant of papillary thyroid carcinoma; FV-PTC, follicular variant of papillary thyroid carcinoma; NIFTP, noninvasive follicular thyroid neoplasm with papillary-like nuclear features; PTC/FTC, mixed papillary thyroid carcinoma and follicular thyroid carcinoma; WDT-UMP, well-differentiated tumor of uncertain malignant potential*.

### *BRAF* Mutation Testing

*BRAF* mutations were detected in 15/199 indeterminate thyroid nodules (8%), and in 12/69 (17%) thyroid malignancies. The classic c.1799T>A (p.V600E) mutation was found in 10/15 indeterminate cases (67%). Two rare *BRAF* mutations were identified: (i) a point c.1801A>G mutation (p.K601E) in 4 patients; and (ii) an in-frame triplet deletion c.1799_1801delTGA (p.V600_K601>E) with no frame-shift in 1 patient (Table 2). The sensitivity, specificity, PPV, and NPV for malignancy of these *BRAF* mutations are given in Table 3.

### *TERT* Promoter Mutation Testing

*TERT* promoter mutations were detected in 7/199 (4%) nodules. There was no overlap between C228T and C250T mutations, the former being more prevalent than the latter. Only 2/7 patients carried an isolated somatic *TERT* promoter mutation, one a C228T and the other a C250T (both FTC-WI). In 5/7 nodules, the *TERT* promoter mutation was associated with another mutation in the *H-, K-, N-RAS*, or *BRAF* oncogenes (Table 2). No *TERT* promoter mutations were found in non-cancerous lesions, so their specificity and PPV for malignancy was 100%, while their sensitivity and NPV were 10 and 68%, respectively (Table 3).

### Combined Molecular Panel

Fine-needle aspiration samples were positive for gene mutations in at least one analysis in 53/199 cases (27%) (Table 3), and 33/53 (62%) of these mutated nodules were malignant. Thirty-three of the 69 malignant nodules carried a mutation, so the panel was able to identify 48% of the malignant lesions. The panel thus achieved a sensitivity, specificity, PPV, and NPV for malignancy of 48, 85, 62, and 75%, respectively, giving a posttest probability of cancer of 62% (PPV) in indeterminate nodules found positive for gene mutations. This corresponds to a 27% improvement in the posttest over the pretest prediction of the cancer risk. For indeterminate nodules proving negative for mutations, on the other hand, the predicted cancer risk decreased from 35% pretest to 25% posttest (Table 3). A distinction between TIR 3A and TIR 3B was available for 111 nodules: in the presence of any gene mutation, the panel revealed a 50% sensitivity, 78% specificity, 37% PPV, and 84% NPV for predicting the cancer risk in the TIR 3A category, and percentages of 39, 85, 79, and 50%, respectively, in the TIR 3B category.

## Discussion

Our objectives in this study were to examine the frequency and significance of *H-, K-*, and *N-RAS, TERT* promoter and *BRAF* gene mutations in a large monocentric series of cytologically indeterminate thyroid nodules and to explore the utility (if any) of such a combined gene mutation analysis in terms of improving indeterminate thyroid nodule characterization prior to surgery.

To date, this is the largest monocentric European series of *RAS*-mutated cytologically indeterminate thyroid nodules with a histological diagnosis. *RAS* mutations were identified in 18% of the indeterminate nodules in our series, and proved to be the most common mutations. This percentage is higher than hitherto reported in patients undergoing thyroid surgery, which has been about 12% in other European series (18, 19), and as low as 6% in American series (7). On the other hand, it was 11.5% when Nikiforov et al. restricted their assessment to patients who underwent surgery with an AUS/FLUS, or with a follicular neoplasm or suspected follicular neoplasm (FN/SFN) cytology (7). Cancer was found in 53% of the *RAS*-mutated nodules in our series, a considerably lower proportion than the 85% reported in American series (7), but much higher than the 18% identified in another recent Italian report (20) involving only 11 *RAS-*mutated patients who underwent surgery, and whose cytologies were mostly benign. On the other hand, the cancer occurrence rate in our *RAS-*mutated nodules was comparable with that of another European series (18), in which the significance of the mutation was analyzed in a similar patient setting.

As for the frequency and significance of *BRAF* mutations in cytologically indeterminate thyroid nodules, the percentage of mutated nodules in our series was 8%, which is slightly higher than reported elsewhere in the literature for indeterminate nodules, i.e., 5% in a recent meta-analysis on nine studies, and 6% in a large individual study published after the meta-analysis (18, 21). So, *BRAF* testing alone has a low sensitivity (17%) in this setting. In addition, the *BRAF* K601E mutation needs to be considered separately because of its low probability of being associated with cancer: 3/4 *BRAF* K601E-mutated nodules in our series were benign. Two of the 3 patients harboring this mutation with a benign histology revealed FA with a microfollicular growth pattern, while the third had a noninvasive follicular thyroid neoplasm with papillary-like nuclear features (NIFTP). These three patients’ slides were reviewed to confirm their histopathological diagnosis. They were not the first cases to be reported of FA associated with a *BRAF* K601E mutation (22, 23), but—to our knowledge—ours is the first report of a *BRAF* K601E mutation occurring in a NIFTP.

This is also the largest monocentric study to have investigated the frequency and significance of *TERT* promoter mutations in indeterminate thyroid FNA samples. With the exception of ThyroSeq. v2 (validated in the setting of FN/SFN cytologies), commercially available molecular panels for thyroid nodules found indeterminate on FNA do not include *TERT* promoter analysis (24). We aimed to ascertain the value of testing *TERT* promoter mutation analysis in a large collection of indeterminate nodules (including cytologies with less aggressive features than those in the FN/SFN category) to see if it might be useful and cost-effective to include it in such commercial panels. In our series, *TERT* mutations were found in 7/199 (4%) FNA samples, and in 7/69 (10%) histologically confirmed malignant nodules. All but two cancers found positive for *TERT* promoter mutations (5/7) also harbored another *RAS* or *BRAF* gene mutation. *TERT* promoter mutation testing alone thus revealed a low diagnostic sensitivity in indeterminate FNA samples, but a 100% specificity. We only found *TERT* promoter mutations in follicular-derived thyroid cancer with a follicular growth pattern—4/7 cases were widely invasive FTC (FTC-WI), and 3/7 were follicular variant of PTC (FV-PTC)—while none of the classical PTC in our series revealed any *TERT* mutations. All the FTCs harboring *TERT* promoter mutations were widely invasive, consistently with the literature correlating these mutations with a more aggressive disease and a worse prognosis. It is worth noting that one patient harboring a *TERT* promoter C250T point mutation also carried a *BRAF* K601E mutation: the association between *TERT* promoter and *BRAF* V600E mutations has been amply described in the literature (13), but ours is the first report (to our knowledge) of an association between *TERT* promoter and *BRAF* K601E mutations. This case seems particularly interesting because it is only the fifth case of FTC associated with a *BRAF* K601E mutation to be reported in the literature (22, 25–27). Three of the five known FTCs with the *BRAF* K601 mutation had minimal capsular and vascular invasion, consistent with the tendency of this mutation to be associated with low-grade cancers (22, 26, 27). The fourth (25) also harbored a *PIK3CA* mutation and the patient had extensive angio-invasion. Our case is therefore the second in which FTC harboring a *BRAF* K601E mutation is associated with extensive vascular invasion. We surmise that the *BRAF* K601E and *TERT* C250T mutations worked in synergy in the pathogenesis of our patient’s FTC: this tumor’s tendency to have more aggressive phenotypic characteristics may be due not only to the presence of the *TERT* promoter mutation, but also to its association with the *BRAF* K601E mutation. Given the strong association between *TERT* promoter and *RAS* mutations, and the case of the former being combined with a *BRAF* K601E mutation, our *TERT* promoter mutation analysis was able to identify only two additional cases of cancer in our series, so it was of little use for cancer identification purposes, in this class of cytologies at least. On the other hand, *RAS* and *K601E* mutations alone carry a low-to-moderate cancer risk (53% for the former and 25% for the latter), so the identification of a *TERT* promoter mutation might still be useful to the clinician in the setting of indeterminate nodules.

Returning to the usefulness of combined molecular panels, their use raised the cancer risk for indeterminate thyroid nodules found positive for genetic mutations to 62% (up 27% with respect to the pretest level), which is similar to the risk associated with TIR 4 lesions. Our PPV of 62% is lower than in an American series (in which it ranged from 87 to 95%), but comparable with the percentage seen in other European series. This disparity may be due to the higher percentage of cancer documented in American *RAS*-mutated indeterminate nodules, possibly because the Bethesda and SIAPEC classifications do not perfectly overlap (7, 18), and/or because our panel did not include *RET/PTC* and *PAX8/PPARG* rearrangement analysis. The introduction of the new histopathologic nomenclature of NIFTP may have played a part too in reducing the PPV of molecular panels for indeterminate lesions, as expected (28). Differences in detection methods could have played a role as well. Histological correlation demonstrated that applying our panel to indeterminate thyroid nodules prompted the identification of 48% of the malignant lesions, an efficiency in identifying cancer that resembles the results achieved by Eszlinger et al. in their monocentric European study (18).

Our study has some limitations to consider. First, it was conducted on a consecutive series of patients referred for surgery, which is probably why their pretest probability of cancer was rather higher than expected (35% vs 25–30%), giving rise to a clinical setting in which “rule-in” molecular tests might be particularly effective. This is certainly not the first series of cytologically indeterminate thyroid nodules with such a high percentage of cancers (29), however. At our institution, FNA cytology is only performed for isolated nodules and those with suspect features on ultrasound, and it is well known that the presence of even one suspicious sonographic feature raises the cancer risk in indeterminate nodules (30). In addition, not all the patients in our series were classified according to the latest SIAPEC classification, and this prevented us from thoroughly examining the utility of molecular analysis in nearly half of our sample, though there was evidence of a markedly higher NPV for cancer in TIR3A than in TIR 3B patients (84 vs 50%). In other words, the utility of the gene mutation panel may be greater when the cancer rate is better defined.

In conclusion, we feel that the outcome of molecular testing, as done in our large series of cytologically indeterminate thyroid nodules, can orient physicians’ and surgeons’ patient management in clinical practice. In the light of our findings, 92 patients could have been spared total thyroidectomy and treated adequately with a lobectomy, and 4 total thyroidectomies would have been performed in one instead of two steps, possibly with fewer side effects. On the other hand, five unnecessary total thyroidectomies would have been performed in patients revealing genetic mutations associated with a benign histology. It is worth adding that there is currently a move toward novel approaches for the management of thyroid nodules in an effort to limit their over-diagnosis and over-treatment. According to the new American Thyroid Association guidelines, thyroid lobectomy suffices for malignant nodules smaller than 4 cm, with no extrathyroidal extension or clinical evidence of lymph node metastases (Recommendation 35) (1). Prospective and controlled studies resulting from the application of these guidelines are still lacking, however. Meanwhile, molecular testing may be useful for stratifying cytologically indeterminate categories for the purpose of deciding on their surgical treatment. The sensitivity of *TERT* promoter mutation for the purpose of predicting cancer risk is low in the indeterminate category, so testing for this mutation may not be cost-effective. In such a particular setting, however, where it remains mandatory to recognize rare cancers with an aggressive behavior, testing for TERT promoter mutations might still prove its worth, particularly in the preoperative setting.

## Author Contributions

SC: study concept and design, substantial contributions to data acquisition, analysis and interpretation, drafting of the manuscript, final approval of the version to be published, and agreement with all aspects of the work; EC: study concept and design, substantial contributions to data acquisition and analysis, drafting of the manuscript, final approval of the version to be published, and agreement with all aspects of the work; SW-F, MI, EI, and FV: substantial contributions to data acquisition and interpretation, critical revision of the manuscript, final approval of the version to be published, and agreement with all aspects of the work; LB, SB, and PL: substantial contributions to data acquisition, analysis and interpretation, critical revision of the manuscript, final approval of the version to be published, and agreement with all aspects of the work; DN: study concept and design, substantial contributions to data acquisition and analysis, final approval of the version to be published, and agreement with all aspects of the work; FG, GP, and AF: substantial contributions to data acquisition, critical revision of the manuscript, final approval of the version to be published, and agreement with all aspects of the work; CM: study concept and design, drafting of the manuscript, critical revision of the manuscript, final approval of the version to be published, and agreement with all aspects of the work, study supervision.

## Conflict of Interest Statement

The authors declare that the research was conducted in the absence of any commercial or financial relationships that could be construed as a potential conflict of interest.
